# Supplementary material for: Are carbon emissions trading and green financial instruments synergistic? -Comprehensive quantitative research based on content analysis
Source: PLoS One. 2024 Mar 7;19(3):e0298601. doi: 10.1371/journal.pone.0298601 (PMC10919601; doi:10.1371/journal.pone.0298601)
Supplement: S1 Data — (ZIP) [file pone.0298601.s001.zip › Data/地级市Z_F工作报告.zip!/╜¡╬≈/╙Ñ╠╢╩╨/└┤╫╘╣1⁄2╓┌║┼í╛┬φ┐╦╩2╛▌═°í┐_2019.docx]

各位代表：

　　现在，我代表市人民政府向大会报告工作，请予审议，并请各位政协委员和列席会议的同志提出意见。

　　一、2018年工作回顾

　　2018年，在省委、省政府和市委的坚强领导下，全市上下深入学习贯彻习近平新时代中国特色社会主义思想和党的十九大精神，从更高层次贯彻落实习近平总书记对江西工作的重要要求，认真落实省委“二十四字”方针，按照市委“三大四聚”发展路径和“六强四动一保障”工作思路，紧扣高质量跨越式发展要求，统筹做好稳增长、促改革、调结构、优生态、惠民生、防风险各项工作，务实重行、砥砺奋进，较好完成了市九届人大四次会议确定的目标任务。全年预计（下同）实现生产总值820亿元、增长8.5%左右，实现财政总收入140.87亿元、增长10.3%，一般公共预算收入80.6亿元、增长7.15%，规模以上工业增加值增长8.6%，固定资产投资增长11%，社会消费品零售总额210亿元、增长10.8%，城镇居民人均可支配收入34232元、增长8%，农村居民人均可支配收入16100元、增长8.8%。

　　一年来，我们主要做了以下工作：

　　（一）着力抓创新，物联鹰潭走在前列。深入推进03专项试点示范基地建设，鹰潭智慧科创城列入省“五大科创城”建设，成功举办创客中国双创大赛和5G应用大赛，获批国家新型工业化产业示范基地，荣获中欧绿色和智慧城市国际大奖。建成国内首个实现中、低两种不同速率移动物联网全域覆盖城市，被中国电信、中国联通列为全国5G试点城市。智联小镇全面启动，中车智慧旅游交通装备生产、中临鹰智能装备产业园、欧菲炬能物联网模组制造基地、浙江沃得尔汽车传感器、普华鹰眼无人机基地、明芳汽车零部件制造等一批重大项目落地建设。物联网研究中心二期工程、华为物联网平台和产业云平台、北航江西研究院鹰潭分院、移动物联网科技成果转化综合服务中心、决策指挥中心、江西先进铜产业研究院、物联网金融创新中心、华北电力大学能源互联网研究中心江西分中心等平台加快推进。物联网终端产品出货量达546.9万件，新增物联网企业102家、总数达198家，产值突破200亿元。主城区智能水表全域覆盖，90%以上智能路灯改造完成，“互联网+交通运输安全”综合监管经验在全国推广，形成智慧党建、“北斗+窄带”地质灾害监测等应用场景30余个。261地质大队、渥泰环保院士工作站挂牌成立，铜产业工程技术研究中心获批国家级博士后科研工作站，新增高新技术企业40家、各类科技创新平台21家，专利申请和授权量增幅、全社会研发投入占GDP比重均居全省前列。

　　（二）着力抓转型，产业升级步伐加快。“2+3”工业体系不断健全，全市规模以上工业主营业务收入增长6%。与江铜集团、江西理工大学、哈尔滨理工大学、江西师范大学等达成战略合作，127个投资亿元以上工业项目加快推进，实施投资500万元以上技改项目85个，完成数字化车间改造35个、智能化生产线改造70条，红旗铜业、凯安智能等12家铜企业入围全省民营企业百强，7家企业获批国家级、省级智能制造试点示范企业，贵溪经开区高端线缆线束生态科技产业集群被评为省级重点工业产业集群。开展闲置低效用地清理专项行动，消化批而未用土地10000亩，新建标准厂房68万平方米。成功承办中国鹰潭·龙虎山道文化旅游峰会、第十届海峡两岸道文化论坛、第六届中部六省炎黄文化论坛、2019海峡两岸道教界迎春联谊会，大上清宫遗址成功入选2017年度全国十大考古新发现，与华侨城集团达成战略合作，“魅力鹰潭·龙虎山”号高铁开通，青茅境景区入选中国森林体验基地，灵溪小镇、渡坊水村分别获批省5A、4A级乡村旅游点，华泉小村、红雨蓝田获评省级旅游风情小镇，鹰潭成为全省唯一一个获评省级全域旅游示范区的设区市，全市旅游接待人次、旅游总收入分别增长31%、33%。我市获批国家级物流枢纽陆港型承载城市、省级城乡高效配送试点城市，市现代物流园跻身国家级示范物流园区，贵溪市荣获全国电子商务进农村综合示范县。粮食总产73万吨，建成高标准农田10.49万亩，新增农业结构调整面积13.6万亩，白鹤湖、杨溪、春涛、锦江现代农业示范园获批省级现代农业示范园，新增各类新型农业经营主体320家，市级以上农业产业化龙头企业新增20家、达255家，产值110亿元、增长8.2%。

　　（三）着力抓改革，发展活力明显增强。“放管服”改革成效明显，市本级388项政务服务事项实现“一次不跑”或“只跑一次”，精简证明材料197项，“互联网+政务服务”全域一体化平台上线，市区两级1480项政务服务事项“一网通办”；实现“41证合一”，企业开办时间压缩至4个工作日，新增各类市场主体1.4万户、增长27%。扎实推进供给侧结构性改革，降成本优环境专项行动为企业减负51.3亿元。组建交建投、工投、财投等国有融资平台，设立市产业投资引导资金，开发区改革创新、市县机构改革、事业单位改革等工作稳步推进，锦江镇列入第一批省级创新型乡镇建设试点。圆满完成余江撤县设区。余江三项国家改革试点工作进入总结阶段，908个规划区外宅改试点村99%完成验收，集体经营性建设用地入市和征地制度改革稳步实施。全域宅改（除余江外）在1613个自然村推开，占规划区外自然村总量82.88%，共退出宅基地14623宗167.78万平方米。农村集体产权制度改革有序推进，自然资源统一确权登记试点工作基本完成。殡葬改革稳步实施，殡葬基础设施建设、“三沿六区”乱埋乱葬清理基本完成。举办鹰潭（深圳）绿色照明产业合作交流会、眼镜产业合作交流会等系列招商活动，全年新增签约项目344个，投资总额903.2亿元，其中投资10亿元以上项目43个，引进省外2000万元以上项目实际进资383.93亿元、增长9%，实际利用外资3.12亿美元、增长8%。中欧(亚)班列开通运营，鹰潭眼镜基地获批国家外贸转型升级基地，连续九年入围中国外贸百强城市。

　　（四）着力抓创建，城乡面貌焕然一新。《鹰潭市城乡总体规划（2015-2030）》暨“多规合一”编制成果正式获批，“一江两岸”城市设计和城市双修规划编制基本完成，城镇化率达60.48%、提高1.4个百分点。“双创”活动全面启动，城乡环境综合整治三年行动和百日攻坚“净化”行动成效显著，顺利通过省级卫生城市复审。鹰西大桥、院里路贯通、“一江两岸”城市景观亮化提升、杏园菜场改造等项目竣工，鹰潭港、花桥水利枢纽、余信贵大道、中心城区取水口迁移及供水工程、雨污水管网改造、小街小巷改造等项目加速推进，获批省“公交城市”试点城市。贵溪市入选全国百强县，贵溪东城区、余江城东新区、月湖新城建设步伐加快。新改建农村公路360公里、农村危桥25座、农村公路安防工程526公里，建成1291个新农村建设村点和22个市级试点村，贵塘公路沿线呈现“五十里贵塘、四十里画廊”的美丽乡村画卷。坚决打好污染防治攻坚战，扎实推进生态文明试验区建设，全力做好中央、省环保督察及长江经济带生态环境保护审计反馈问题的整改落实，一批历史遗留的突出问题得到有效解决，全市环境空气质量优良率达88.2%，PM2.5浓度全省第三低，PM10浓度全省最低。 完成生活垃圾一体化处理，城市、农村生活垃圾无害化处理率分别达100%、95%。深入落实河湖长制、林长制，扎实开展“清河行动”，85%以上规模养猪场完成标准化改造，全市主要河流断面水质和集中式饮用水水源水质达标率均为100%。

　　（五）着力抓共享，群众福祉日益提升。省下达的民生指标基本完成，民生支出占财政总支出比重达71.4%，获评2018年中国地级市民生发展百强城市。全力以赴打好脱贫攻坚战，接续开展了脱贫攻坚春季攻势、夏季整改、秋冬会战，创新推广数据比对法、增收激励法、智慧扶贫等模式，脱贫攻坚数据质量位居全省第一，扶贫产业基地、产业扶贫实现全覆盖，1.24万人次享受“六道医疗保障线”政策，获批全国“互联网+健康扶贫”应用试点建设单位，全年预脱贫1740户4824人，预退出7个省级贫困村和3个市级贫困村。积极做好城镇贫困群众脱贫解困工作。新增城镇就业2.53万人，新增转移农村劳动力1.36万人，城镇登记失业率控制在4.5%以内。扎实推进全民参保，社会保障卡基本实现全市“人手一卡”。2018年各类棚改住房开工建设7778套，农村危房改造1027户，均完成省下达任务。实施公积金按月对冲还贷等政策，房地产去库存成效明显。大力推进各类教育优质均衡发展，余江义务教育均衡发展通过国家评估验收，全市整体实现县域义务教育发展基本均衡，市红旗干部学院开学，鹰潭应用工程学校迁建、市老年大学等项目有序推进。全市12家二级以上公立医院综合改革成效初显，31家定点医院药店实现跨省异地就医即时结算，市县乡村四级分级诊疗体系初步形成。市美术馆新馆建成使用。举办庆祝改革开放40周年系列活动。央视《银河之声》走进鹰潭。中国血防纪念馆揭牌开馆。樟坪畲族村获评全国生态文化村。第十五届省运会我市奖牌总数、团体总分均创历史新高，举办全国沙滩排球邀请赛、中国乒乓球协会会员联赛鹰潭分站赛、万人太极拳展示等体育活动。龙虎山舰正式命名入列。全面开展扫黑除恶专项斗争，公众安全感大幅提升，荣获全省“平安杯”，6个村（社区）荣获全国民主法治示范村（社区）。开展全省规模最大的森林防火综合实战演练。全域推动“双安双创”，做好非洲猪瘟防控工作，安全生产形势平稳，社会保持和谐安定。妇女、未成年人、老年人、残疾人权益得到保障，新闻出版、广播电视、统计、审计、地方志等工作得到加强，对台、外侨、气象、档案、防空防灾等工作取得新进展。

　　（六）着力抓效能，自身建设不断加强。扎实推进“两学一做”学习教育常态化制度化，严格落实中央八项规定及实施细则精神，深入推进干部作风大转变教育整治活动，认真落实“严、细、实、快、廉”要求，坚决纠正“怕、慢、假、庸、散”等作风顽疾，在全市政府系统开展忠诚型、创新型、担当型、服务型、过硬型政府建设，着力打造“四最”营商环境。扎实推进依法行政，健全完善重大事项依法决策机制，加强政府立法工作，《鹰潭市信江饮用水水源保护条例》颁布施行。自觉接受市人大及其常委会的法律监督、工作监督，主动接受市政协的民主监督和社会舆论监督，办理人大代表建议58件、政协提案153件。压实全面从严治党主体责任，坚决全面彻底肃清苏荣案余毒，深刻汲取李贻煌等腐败案教训，严肃党内政治生活，廉政建设和反腐败斗争不断加强。

　　各位代表，奋斗历经艰辛，成绩来之不易。这些成绩的取得，是习近平新时代中国特色社会主义思想特别是习近平总书记对江西工作的重要要求科学指引的结果，是省委、省政府和市委坚强领导的结果，是市人大、市政协监督支持的结果，是全市上下共同努力的结果。在此，我谨代表市人民政府，向在各个岗位上贡献智慧、付出心血和汗水的全市人民，向各民主党派、工商联、无党派和社会各界人士，向驻鹰单位、人民解放军、武警部队官兵和离退休老同志，表示衷心的感谢，并致以崇高的敬意！

　　需向代表说明的是，受新的经济下行压力等因素影响，固定资产投资、社会消费品零售总额等指标略低于预期目标。同时，我们也清醒看到，工作中还存在不少困难和问题，主要是：传统产业转型升级力度还需加大，实体经济面临的困难仍然不小；新动能支撑不够，创新能力不强、高层次人才不足、体制机制不优、大项目不多等问题比较突出；发展不平衡不充分，中心城区辐射带动能力有限，生态环保压力加大，基本公共服务供给能力不足，与人民群众对美好生活的期待还有很大差距；运用新发展理念创新性开展工作的能力和水平还不高，工作效能和效率有待进一步提升等等。对此,我们一定坚持问题导向、目标导向，采取有效措施，切实加以解决。

　　二、2019年主要工作

　　2019年是新中国成立70周年，是全面建成小康社会关键之年，做好政府各项工作至关重要。今年政府工作的总体要求是：以习近平新时代中国特色社会主义思想为指导，深入贯彻党的十九大、十九届二中、三中全会、中央经济工作会议和省委十四届六次、七次全会精神，从更高层次贯彻落实习近平总书记对江西工作的重要要求，坚持稳中求进工作总基调，坚持新发展理念，坚持高质量跨越式发展首要战略，坚持以深化供给侧结构性改革为主线，深入落实市委“三大四聚”发展路径和“六强四动一保障”工作思路，全力打好三大攻坚战，全力加快产业升级，全力深化改革开放，全力推动城乡融合，全力提升绿色发展，全力保障改善民生，加快推进“小市大为、跨越赶超”，奋力迈出打造“两都一城”、建设富裕美丽幸福现代化鹰潭的坚实步伐，为与全国同步全面建成小康社会收官打下决定性基础，以优异成绩庆祝新中国成立70周年。

　　今年发展的主要预期目标是：生产总值增长8.5%，财政总收入增长7%，一般公共预算收入增长5%，规模以上工业增加值增长8.5%左右，固定资产投资增长10%左右，社会消费品零售总额增长11%左右，城镇居民人均可支配收入增长8.1%，农村居民人均可支配收入增长8.9%，居民消费价格总水平涨幅控制在3%以内，完成省下达的节能减排计划任务。围绕实现上述目标，重点做好五个方面工作：

　　(一)写好“创新篇”，让物联鹰潭的品牌更加响亮。

　　坚持产业为本。加快推进智联小镇建设，产业综合服务中心基本建成，12条道路以及35万平方米定制厂房和标准厂房投入使用，力争中车智慧旅游交通装备生产项目开工投产，中临鹰智能装备产业园竣工运营，着力构建集智慧大健康、智能家居、智慧芯片、智能装备制造四大产业组团于一体的万物智联生态圈。加快贵溪创新创业产业园和余江电子信息产业园、智能穿戴产业园建设，推动欧菲炬能物联网模组、浙江沃得尔汽车传感器、永威光电物联网摄像头、鹰视觉火苗探测传感器等项目扩产增效，培育和引进一批移动物联网硬件制造龙头企业，力争新增物联网企业100家、总数达250家以上，其中制造类企业40家、总数达120家以上，新增物联网产品40款、总数达120款以上，物联网核心及关联产业主营业务收入突破400亿元，物联网产业集群列入国家级战略性新兴产业集群。

　　坚持应用为先。完成重点区域5G网络覆盖，建成70个5G基站，积极推进5G试商用。持续深化十大城市级应用，完成全市智能水表改造、主次干道灯具和城区停车智能改造，推进智慧环保+水利、智慧党建二期、智慧医疗、智慧教育、智慧旅游、智慧物流、智慧消防等应用建设。大力推广已列入省政府协议供货目录的应用示范及成熟产品，推动智慧水表、智能净水设备等成熟产品“走出去”，加快抢占物联网智能终端市场。

　　坚持平台为重。积极创建国家级数字经济示范区，冲刺巴塞罗那全球智慧城市博览会金奖城市。加速提升物联网研究中心、华为（江西）物联网云计算创新中心、移动物联网科技成果转化综合服务中心、决策指挥中心等平台服务能力，加快推进北航研究院鹰潭分院、江西先进铜产业研究院、铜产业大数据中心、铜精深加工应用移动物联网技术协同创新研究院、高端线束线缆产业研究院、华北电力大学能源互联网研究中心江西分中心等产学研平台建设，建成服务全省的产品研发、测试认证、技术支撑、成果转化、产业云服务等平台服务体系。

　　坚持科技为要。深入实施科技创新“四个一”升级工程计划，健全产学研用创新体系，加大规模以上企业研发投入，力争全社会研发投入占GDP比重保持4%以上。深入实施“鹰才计划、乡贤回归”行动计划，引进培育一批领军人才、高端人才和创新创业团队，筹建移动物联网技术学院，加快培养信息通信领域高素质技能人才。深化与知名企业、大院大所、高等院校合作，扩大博士后科研工作站和创新实践基地规模，新增市级以上科技创新平台20家以上。

　　各位代表，物联网正在开启一个新时代，拥抱物联网就能拥有未来。我们要以03专项试点示范基地为契机，驰而不息，持之以恒，久久为功，努力实现从“探路者”到“抢滩者”、从“先行者”到“引领者”的转变，在“生万物”的大地上，早日进入“联万物”的新时代！

　　（二）聚焦“高质量”，让转型升级的步伐更加有力。

　　实施工业三年倍增行动计划。优化产业布局，着力构建“2+3”工业体系，加快构筑“一心带一轴、双线齐联动”的产业空间格局。大力开展铜产业优化升级专项行动，策应江铜集团“创新倍增”战略，继续深化服务江铜发展的24项举措，与江铜在高端线缆产业园共建、银珠山矿开发等方面深化合作，推进鑫铂瑞科技3万吨电子铜箔、云泰铜业10万吨高性能高精度铜板带等38个重点铜项目建设，重新组建铜检中心，加快以区块链技术推动铜期货交割仓库智能化改造，加快中航信托铜物贸市场建设，筹建铜物资储备仓库，力争铜企业智能化改造达50%以上。大力开展特色产业提升专项行动，加快雅视光学眼镜高新配套科技园、晶威眼镜产业园、明芳汽车零部件制造、美智光电智能家居照明、雕刻创意文化集聚区、装备式建筑产业园等项目建设，推动十大乡镇产业集中区建设。大力开展“两型三化”管理提标提档、集群式项目满园扩园行动，实施亩产效益综合评价工作，推进园区智慧化建设，新建标准厂房150万平方米，全市园区消化批而未用土地6000亩，促进开发区体制机制创新。大力开展降成本优环境专项行动，全面落实国家减税降费政策，激发和保护企业家精神，助力民营企业翻越市场的冰山、融资的高山、转型的火山，让民营经济加快走向更加广阔的舞台。

　　实施服务业优化提速发展三年攻坚行动。加快实施旅游景区优质旅游先锋行动三年计划，深化与华侨城集团合作，实施龙虎山景区核心区域改造提升，精心打造龚资线风情廊道等五个片区，推进青茅境、血防红旗博览区等创建国家4A级旅游景区和南山小镇创建省5A级乡村旅游点，积极创建国家级全域旅游示范区、全国森林旅游示范市，突破入境旅游短板，全市旅游接待人次、旅游总收入分别增长25%、35%以上。围绕打造中华道都，推进大上清宫遗址保护性设施建设工程，加快龙虎山道博园、天师林、道学院、道文化主题雕塑公园等项目建设。力争开工建设中国邮政鹰潭邮件处理及仓储中心，建成运营林安物流园，争取列入国家城乡高效配送试点城市。大力引进金融机构，加快物联网金融创新中心、智慧金融产业园建设，加大铜产业供应链金融推进力度，推进企业上市“映山红”行动，建成智慧金融综合服务平台。

　　实施现代农业发展三年专项行动。推动藏粮于地、藏粮于技落实落地，稳定粮食生产能力，建成高标准农田4.37万亩。大力实施农业结构调整行动计划“1+9”方案和林下经济发展行动计划“1+6”方案，“三线四园”建成3万亩现代农业产业示范基地，建成3个省级现代农业示范园，新增200家以上新型农业经营主体。大力发展休闲、观光、康养农业及“互联网+”等新型业态，推动一二三产业深度融合。加快农村土地所有权、承包权、经营权分置改革，完成农村集体产权制度改革中央试点工作。大力发展村级集体经济，确保再消除20%空壳村，再培育一批经济强村。

　　各位代表，面对产业发展实力不强、结构不优、质量不高的现实问题，转型发展、高质量发展、跨越式发展是我们的不懈追求。我们唯有紧紧咬住“产业发展强起来”，不断延伸产业链、拓宽产业幅、打造产业群，才能确保鹰潭在奔小康路上不掉队，在新时代面前不落伍！

　　（三）打好“绿色牌”，让美丽鹰潭的魅力更加彰显。

　　全力开展“双创双修”行动。实施城市功能与品质提升三年行动，深入开展“八大行动”，争取“双修”修出美、“双创”创出质。做强“畅”文章，完成鹰雄大道、鹰南大道等路景改造提升工程，加快推进余信贵大道、鹰潭大桥拆旧建新等项目，实施206国道龙虎山段改造、鹰南收费站改建，开工建设鹰潭港中童、九牛滩作业区货运码头，加快鹰南公交枢纽站、公交调度指挥中心建设。做足“修”文章，加快东湖片区、磷肥厂片区、肉联厂、老玻璃厂、朱埠刘家和信江新区流洪村等片区房屋征收工作，结合棚户区改造和城区重要节点提升，分步推进梅园大道、胜利路、铜兴路、龙虎山大道综合改造提升，打通上清路，加快市二中、市一小等校园周边环境改造，渠化五道口、鹰潭大桥路口、客运站路口等交叉路口，新改建军民路、莲花路农贸市场和主城区73个公厕。做活“水”文章，加快海绵城市建设，实施信江新区引水入城、信江新区中央公园等工程，推进童家河、东川河、白露河流域整治，整体改造老城区和信江新区雨污水管网，完成中心城区取水口迁移及供水工程，逐步建成“河畅、水清、岸绿、景美”的水生态文明城市。做好“创”文章，积极推行下沉式管理、“路长制”，提升市民健康教育、“创卫”宣传、农村卫生健康工作水平，全面掀起“双创”工作热潮。

　　全力实施乡村振兴战略。按照“四精”理念，深入实施农村人居环境整治三年行动，大力开展“六统·四联创”，加快塘湾古镇、锦江古镇、文坊车家古村等特色镇村建设，打造美丽示范乡镇2个、美丽示范村庄20个、美丽示范庭院200个，建设1200个左右新农村建设村点。完成全域宅改推广推进任务，总结提炼余江三年宅改工作经验，开展宅改“回头看”行动，形成制度固化宅改成果。稳妥推进殡葬改革，农村公益性公墓建设全面完成，公益性公墓节地生态安葬率达到90%以上，“三沿六区”散埋乱葬整治全面落实，基本形成现代文明丧葬新风。加快“四好农村路”建设，推进贵溪市全国城乡交通运输一体化试点达标，新改建农村公路500公里、农村危桥15座、农村公路安防工程500公里。

　　全力打好污染防治攻坚战。认真做好中央环保督察、省环保督查、长江经济带生态环境保护审计、中央环保督察“回头看”等反馈问题整改工作，坚决防止环境污染问题反弹回潮。打好蓝天保卫战，聚焦“三尘、三烧、三气”，深入实施燃煤污染、工业废气、城市扬尘、禁燃禁放烟花爆竹等专项治理行动，全市环境空气质量优良率达91.5%，确保全市PM2.5平均浓度控制在35微克/立方米以内。打好碧水保卫战，全面推行河湖长制，继续推进“清河行动”，完成限养区内畜禽养殖场生态化达标改造任务和余江区畜禽粪污资源化利用整县推进项目建设，梯次开展农村生活污水治理，全市主要河流断面水质和集中式饮用水水源水质达标率保持100%。打好净土保卫战，建设全市土壤污染防治项目库，加快推进市静脉智园建设，基本完成生活垃圾焚烧发电厂二期和建筑垃圾消纳场建设，开展垃圾分类试点。实施生态环境修复工程，加大对重要生态保护区、水源涵养区、江河源头区的生态修复和保护力度，全面推行林长制，推进重点区域森林绿化美化彩化珍贵化建设，完成17.8万亩补改造林任务。稳步推进第二次全国污染源普查和第三次全国国土调查工作。实施生态环境损害赔偿制度改革。

　　各位代表，城市让生活更美好，乡村让城市更向往。我们要坚持不懈地把习近平生态文明思想贯穿鹰潭经济社会发展始终，把城市建设当成家庭装修，像保护眼睛一样保护环境，努力让生态成为城市的常态、让绿色成为鹰潭的底色！

　　（四）扛起“改革旗”，让体制机制的优势更加凸显。

　　坚持以改革焕发新动能。纵深推进“放管服”改革，拓展“互联网＋政务服务”全域一体化平台应用，市县两级依申请类政务服务事项“一次不跑”“只跑一次”办理率达70%，网上可办率不低于70%，市县“一窗式”受理率达70%以上，直接为企业和群众提供与日常生产生活密切相关事项的办理窗口实行全天候服务，着力疏解群众办事“堵点”问题。推进“证照分离”改革，实行“多证合一、一照一码”，实现企业登记全程电子化，全面实施“双随机、一公开”市场监管。推进工程建设项目审批制度改革，开展“四联合”改革，实施“三书合一”，实现政府和企业投资项目全流程审批时间分别压缩至79个、60个工作日以内。推动房产交易和不动产登记一体化改革，大力推进“互联网+不动产登记”。完成市县机构改革工作，扎实推进公路建设养护管理体制改革，稳步推进事业单位改革、经济发达镇行政管理体制改革，深化财税、价格、供销等体制改革。继续打好防范化解重大风险攻坚战，稳妥处置地方政府债务风险，加大防范处置非法集资力度。

　　坚持以开放催生新动能。对接“一圈两轴三区”重大区域部署，加速融入大南昌都市圈、沪昆高铁经济带，进一步深化赣东北对外开放合作，逐步建成赣浙闽开放合作重点区域。开展“三请三回”和“三企”入鹰活动，推动首位度产业招商、商务协作小分队专职招商、产业基金招商，着力引进一批龙头型、引爆型、补链型项目，确保国家级开发区至少引进一个投资超50亿元的产业项目，省级开发区至少引进一个投资超20亿元的产业项目，全年引进省外2000万元以上项目实际进资414.65亿元、增长8%，实际利用外资3.28亿美元、增长5%。主动对接“一带一路”，引导优势企业产能出海、境外投资，组织企业参加境内外展会100家（次），加快建设鹰潭保税仓库，实现多线路、多口岸并行的中欧（亚）班列发展目标，力争外贸实现稳中提质。

　　坚持以项目积蓄新动能。坚持以大投入带动大建设促进大发展，开展“大干项目年”活动，初步安排大中型项目387个，年度计划投资574.3亿元，新开工建设179个、建成134个。重点抓好普华鹰眼无人机、嘉华乐器等164个重大产业项目，花桥水利枢纽工程、贵溪至资溪高速公路、G320贵溪段南迁等117个重大基础设施项目，城南污水处理厂提标扩容及污泥处理厂、海棠公园等21个生态环保项目，鹰潭应用工程学校迁建、市老年大学等62个重大民生和社会事业项目。继续抓好鹰潭通用机场、鹰潭至鄱阳城际铁路等项目前期工作，谋划储备一批重大项目。发挥投资公司、工投、交建投、财投等投融资平台作用，提高运营质量和效益。

　　各位代表，改革开放只有进行时，没有完成时。鹰潭经济正处于新旧动能转换的关键期，我们要抢抓发展机遇期和政策窗口期，用好市场化改革、高水平开放这个“金钥匙”，扭住大干项目、干大项目这个“牛鼻子”，实现新旧动能接续转换、双轮驱动，让发展调速不减势、量增质更优！

　　（五）织好“幸福网”，让人民群众的生活更加美好。

　　让老百姓有现实的获得感。深入实施打赢脱贫攻坚战三年行动，高质量推进十大扶贫工程，大力推广“一领办三参与”产业扶贫新模式，让有劳动能力的贫困户家家有就业，让特殊群体贫困户人人有保障。建立稳定脱贫的长效机制，完善贫困人口动态管理机制，加大扶志扶智扶勤扶德力度，确保遇困、遇病、遇灾不返贫，确保全年完成脱贫1997户4929人，退出1个省级贫困村和4个市级贫困村。实施城镇贫困群众脱贫解困工作，完成脱贫解困6986人。坚持就业优先战略和积极就业政策，引导促进更多高校毕业生来鹰返鹰创业就业，城镇新增就业1.96万人，新增转移农村劳动力1.25万人。精准深入实施全民参保计划，实施社会保障卡“一卡通”二期工程，稳步提高城乡基本养老、基本医保、城乡低保等补助标准，推进基本医保跨省异地就医住院费用直接结算。加快构建房地产市场健康发展长效机制，完善住房市场体系和保障体系。

　　让老百姓有直观的幸福感。实施第三期学前教育行动计划、普通高中阶段教育普及攻坚计划、第二期特殊教育提升计划，推进义务教育由基本均衡向优质均衡发展，加快中小学生示范性综合实践基地、市实验中学重建、高桥中学、信江新区九年一贯制学校、周塘九年一贯制学校等项目建设，推进校长教师交流轮岗，推动江西师专更好服务经济发展，深化职业教育产教融合。继续推进公立医院综合改革，完善分级诊疗、现代医院管理、全民医疗保障、药品供应保障、综合监管等5项制度，深化“互联网+健康扶贫”应用试点建设，加快市妇幼保健院和市中心血站异地新建、月湖区人民医院等项目建设。组织开展庆祝新中国成立70周年文化艺术创作和展览展演活动，加快“四馆”、角山遗址保护工程建设，推进公共文化设施免费开放。支持社会资本设立养老机构，建成运营月湖湾颐养园，加快智慧养老和智慧社区建设，全市居家养老点达278个。促进体育、新闻出版、广播电视、档案、地方志等事业发展，发展防空防灾、气象、残疾人事业，做好民族、宗教、外事、侨务等工作，支持工会、共青团、妇联等群团组织和社会组织履行职责。

　　让老百姓有充分的安全感。深化“打造全省治安首善之地”活动，深入推进扫黑除恶专项斗争，加快“雪亮工程”建设，建立网格化社会治理模式，健全社会公共安全立体防控、防灾减灾和应急救援体系，实施“法律明白人”培养工程，提升市公共法律服务实体平台服务能力，打造“指尖上的信访”，推进信访法治化建设。支持服务深化国防和军队改革，加强国防教育、优抚安置和双拥共建，积极争创全国、省级“双拥模范城”八连冠，做好“鹰潭舰”命名申报工作。深入推进“双安双创”工作，完善智慧市场监管网格化管理模式，建成使用新畜禽屠宰厂，打造国家级产品质量提升示范区。加强安全隐患整治，完善安全生产责任体系。

　　各位代表，民生是发展的目的，民心是最大的政治。无论今年财政压力有多大，民生投入只多不少，社会事业支出只增不降，为群众办的实事只加不减，让老百姓的获得感、幸福感、安全感在家门口升级，努力让孩子们快乐成长、年轻人创业打拼、老年人颐养天年，让全市人民生活得更加幸福、更有尊严！

　　三、努力建设人民满意的“五型”政府

　　进入新时代，开启新征程，我们将不忘初心、牢记使命，以真抓的实劲、敢抓的狠劲、善抓的巧劲、常抓的韧劲，真正顶起自己该顶的那片天，努力打造能够适应新时代、彰显新气象、实现新作为的“五型”政府。

　　（一）强化政治抓落实，建设忠诚型政府。坚持把政治标准摆在“五型”政府建设的首位，深入学习贯彻习近平新时代中国特色社会主义思想和党的十九大精神，树牢“四个意识”，坚定“四个自信”，坚决做到“两个维护”，任何时候、任何情况下都始终保持对以习近平同志为核心的党中央的绝对忠诚。坚决贯彻落实党中央国务院、省委省政府和市委的各项决策部署，始终做到在大局中定位、在大局中谋划、在大局中工作，让旗帜鲜明地真讲政治、实讲政治、严讲政治在全市政府系统中蔚然成风。

　　（二）强化本领抓落实，建设创新型政府。深入学习以习近平同志为核心的党中央治国理政新理念新思想新战略，及时充电充实新技术、新业态、新模式、新知识，提升专业素养，增强服务本领，坚决杜绝“身子进入新时代，脑子还停留在过去式”。围绕“六个强起来”，不断研究新情况、探索新方法、总结新经验、解决新问题，进一步增强区域聚焦、产业聚焦、政策聚焦、资源聚焦的操作力和执行力。加快实施大数据治理体系建设，强化政府系统资源整合、力量打通、流程再造、数据共享，推进政府治理数字化转型。

　　（三）强化责任抓落实，建设担当型政府。实行“即决策、即执行、即监督、即反馈”的高效闭环工作机制，推行项目落地工作法和政府工作清单管理、挂图作战、擂台比拼，大力倡导“事事马上办、人人钉钉子、个个敢担当”和“不为不办找理由，只为办好想办法”，确保政府工作掷地有声、落地生根。改善和规范督查工作，最大限度削减“文山会海”“材料报表”，下气力解决重“迹”不重“绩”、留“绩”不留“心”问题，把干部从无谓的事务中解脱出来，把更多时间和精力用在抓落实上。实施容错免责机制，鼓励干部勇于担当、积极作为。

　　（四）强化宗旨抓落实，建设服务型政府。强化服务意识，营造“四最”营商环境，构建亲清新型政商关系，吸纳八方客商，激发创业活力，在“零障碍”上求突破，在低成本上下功夫，在高效率上出实招。坚持以人民为中心的发展思想，从人民群众关心的事情做起，从让人民群众满意的事情做起，对接“问政追踪”“鹰眼追踪”等专栏，多进“施工地”，多钻“矛盾窝”，高质量履行经济调节、市场监管、社会管理、公共服务、生态环境保护等职责。

　　（五）强化作风抓落实，建设过硬型政府。坚持将政府工作纳入法治化轨道，自觉接受党内监督、人大监督和政协民主监督，广泛听取民主党派、工商联、人民团体等各界意见，主动接受司法监督、群众监督、舆论监督，让权力在阳光下运行。严格落实全面从严治党主体责任，全面彻底肃清苏荣案余毒，高标准贯彻中央八项规定及实施细则精神，聚焦“怕、慢、假、庸、散”等作风顽疾，把群众的意见作为“最好的镜子”，严惩侵害群众利益的不正之风和害群之马，严治“雁过拔毛”式的微腐败，切实把“严、细、实、快、廉”的新风正气树起来。

　　各位代表，事业都是打拼出来的，幸福都是奋斗出来的。让我们更加紧密地团结在以习近平同志为核心的党中央周围，在习近平新时代中国特色社会主义思想的指引下，在省委、省政府和市委的坚强领导下，聚全民之力、扬奋进之帆，当好新时代的答卷人，加快推进“小市大为、跨越赶超”，奋力迈出打造“两都一城”、建设富裕美丽幸福现代化鹰潭的新步伐，以优异成绩庆祝新中国成立70周年！
